# Survey of the fecal microbiota of indigenous small ruminants living in different areas of Guizhou

**DOI:** 10.3389/fmicb.2024.1415230

**Published:** 2024-08-08

**Authors:** Wei Guo, Tingmei Liu, Weiwei Wang, Yinshu Yu, André Luis Alves Neves, Mi Zhou, Xiang Chen

**Affiliations:** ^1^Key Laboratory of Animal Genetics, Breeding and Reproduction in the Plateau Mountainous Region, Ministry of Education, College of Animal Science, Guizhou University, Guiyang, China; ^2^Department of Agricultural, Food and Nutritional Science, University of Alberta, Edmonton, AB, Canada; ^3^Department of Veterinary and Animal Sciences, Faculty of Health and Medical Sciences, University of Copenhagen, Frederiksberg, Denmark

**Keywords:** fecal microbiota, goat, sheep, enterotypes, Guizhou

## Abstract

**Introduction:**

Gut microbiota are associated with the health and performance of ruminant species, and they are affected by altitude, host genetics, and sex. However, there has been little research on comparing the fecal microbiota of indigenous small ruminants such as sheep and goats in Guizhou province, China. In the present study, we revealed the effect of altitude, genetics, and sex on fecal microbiota profiles and enterotypes in indigenous small ruminants of Guizhou province, China.

**Methods:**

Fecal samples were collected from Hei and Qianbei Ma goats and Weining sheep in the Chinese province of Guizhou. 16S rRNA gene sequencing targeting the V3–V4 region was performed using the Illumina MiSeq platform. Sequences were processed using QIIME2, and the qualified sequences were processed using the plugin DADA2 to generate amplicon sequence variants (ASVs). The statistical analysis was performed using R studio.

**Results:**

The fecal microbial profile was found to vary by herd (influenced by genetics/altitude) and sex. All samples were categorized into two enterotypes. The first enterotype is dominated by *UCG-005*, and the second enterotype is dominated by the *Christensenellaceae_R-7_group*, which may be highly driven by the host's genetics (breed). The predicted functional profiles of the fecal microbiota were also assigned to two clusters that corresponded exactly to the enterotypes. Cluster 1 of the functional profiling was characterized by biosynthesis pathways, and cluster 2 was characterized by energy metabolism pathways.

**Discussion:**

Our findings may provide new insights into the fecal microbial community and enterotypes in small ruminants by herds, offering clues for understanding the mechanisms by which the fecal microbiota contribute to divergent host phenotypes in indigenous small ruminants in Guizhou.

## Introduction

In southwest China, the altitude of Guizhou province ranges from 200 m (east) to 2,800 m (west), and it is considered the Guizhou Plateau. Multiple domestic animal species, such as cattle, sheep, and goats, are pastured in this area by farmers. In 2022, the ruminant population in Guizhou was 875 million (with cattle accounting for 485 million and sheep and goats making up 390 million), contributing to approximately 3% of the local agricultural output. Among them, small ruminants raised in this area contribute to a large proportion of the livestock industry (5%), and are thus of great economic value.

It has been shown that the composition of gut microbiota is influenced by common factors including genetics, sex, and altitude (Moeller and Sanders, 2020; Bai et al., 2022; Guo et al., 2022). In addition, rumen microbiota are essential for converting recalcitrant plant materials into nutrients for the host and for maintaining bovine health (Lopes et al., 2019; Virgínio Júnior and Bittar, 2021), while fecal microbiota are indicators of hindgut microbiota, which are involved in the last stage of plant material digestion and absorption and in supporting host health (Mao et al., 2015; Guo et al., 2020; Wang et al., 2023). Therefore, the impact of altitude, genetics, and sex on rumen and fecal microbiota of ruminants has attracted increasing attention. Recent studies have suggested that the fecal microbiome and metabolomics of Sanhe heifers (Zhang et al., 2023), as well as rumen microbiota composition and fermentation profiles, change with altitudes (Han et al., 2022). In addition, the composition and function of the fecal microbiota in wild blue sheep vary with sex (Zhu et al., 2020), as do the composition and fermentation profiles of rumen microbiota (Guo et al., 2022). Furthermore, the evenness of fecal bacterial (Mahayri et al., 2022) and the composition of rumen bacteria (Wu et al., 2020) are affected by host genetics. These results indicate that altitude, host genetics, and sex are important factors that can affect the diversity and composition of gut microbiota. Although some studies have been conducted to explore the rumen microbiota community of local goats in Guizhou (Tian et al., 2021, 2022; Zhou et al., 2022), there has been little research on comparing the fecal microbiota of indigenous small ruminants such as sheep and goats in Guizhou province, China.

In the current study, we collected fecal samples from three individual herds consisting of two goat breeds and one sheep breed living between 1,000 and 2,200 m above sea level (a.s.l) in a high-mountain ecosystem. We included both male and female animals from each population to determine the effect of altitude, genetics, and sex on fecal microbiota profiles and enterotypes in small ruminants.

## Materials and methods

### Animals and sampling

The sheep and goats used in this study were aged between 2 and 4 years, and the experiment was conducted between July and August 2022. In this study, 35 fecal samples were collected from Weining sheep (H) (F = 23 and M = 12), 23 from Qianbei Ma goats (L) (F = 11 and M = 12), and 32 from Hei goats (M) (F = 16 and M = 16), raised in Weining (26°36′N, 103°36′E; 2,200 m), Xishui (28°19′N, 106°12′E; 1,040 m), and Weining (26°44′N, 104°39′E; 1,900 m), respectively, in Guizhou, China. The Weining sheep (a dual-purpose breed for wool and meat) and Hei goats (meat) grazed freely in the natural pasture, and the dominant and associated plant genera included *Digitaria, Poa, Agrostis*, and *Festuca*. The Weining sheep and Hei goats were corralled the night before the sample collection. The Qianbei Ma goats (a dual-purpose breed for wool and meat) were housed indoors and fed a basal diet twice daily (9:00 and 17:00), with free access to water, as described in a previous study (Fu et al., 2022), and weaned at 3 months of age. Before the morning feeding and grazing, the fecal samples were collected from the rectum of the animals using clean disposable latex gloves and were frozen in liquid nitrogen immediately and then stored at −80°C until further analysis.

### DNA extraction and amplicon sequencing

Total genomic DNA was extracted from the fecal samples using a QIAamp DNA stool mini kit (Qiagen, Valencia, CA, US) following the manufacturer's instructions. The concentration of the extracted DNA was checked using a Nanodrop spectrophotometer (Thermo Scientific, Wilmington, DE, US). The qualified DNA was then amplified using the primer set 338F/806R targeting the hypervariable V3–V4 regions of bacterial 16S rRNA following standard PCR conditions (Kim et al., 2021). The qualified PCR products were sequenced using the Illumina MiSeq platform (2 × 300 paired-end sequencing runs). All the data used in this study are available in the National Center for Biotechnology Information Sequence Read Archive (NCBI SRA) under the accession number PRJNA1006184.

### Bioinformatics and statistical analysis

Sequences were processed using QIIME2 (v 2022.2) (Bolyen et al., 2019). After demultiplexing, the sequences were quality filtered, the chimera was removed, and the subsequent reads were merged using the plugin DADA2 to generate amplicon sequence variants (ASVs) (Callahan et al., 2016). Then, a taxonomic classification of the ASVs was obtained using the plugin q2-feature-classifier against the SILVA 132 99% reference database (Kim et al., 2021). For diversity analysis, the sequences were rarefied to 16,006 reads per sample to avoid uneven sequencing depth. Furthermore, the alpha diversity was determined using Shannon and Chao1 indices, while the beta diversity was estimated based on the Bray–Curtis distances, which was then presented in a principal coordinate analysis (PCoA) plot. Finally, microbial functional predictions and pathway inferences were performed using PICRUSt2 based on the MetaCyc database (Caicedo et al., 2020). The statistical data analysis was conducted using R studio (version 3.5.3). For example, the differential abundance of the taxonomic composition (at the phylum and genus levels) and the Metacyc pathways among herds were determined by conducting linear discriminant analysis effect size (LEfSe) (Segata et al., 2011). A differential analysis of the alpha diversity among herds was conducted using the Kruskal–Wallis test followed by Dunn's *post hoc* multiple-comparison text. A Mann–Whitney U test was performed to compare the median similarities of the alpha diversity between female and male animals within each herd. Differences in the microbial composition within each herd were calculated using DESeq2 (Love et al., 2014). The effect of herd and sex on the beta diversity was evaluated using PERMANOVA with the default parameter (Anderson, 2014). Taxa that occurred in at least 25% of the animals within each herd and had a relative abundance of >0.1% were retained for the subsequent analysis.

Enterotype clusters at the genus level were calculated using the Jensen–Shannon divergence (JSD) distance and the Partitioning Around Medoids (PAM) clustering algorithm (Mobeen et al., 2018). The optimal number of clusters was determined using the Calinski–Harabasz (CH) index, while the robustness of the clusters was evaluated using the Silhouette index (Mobeen et al., 2018). The Mann–Whitney U test was employed to determine the differential microbial taxa between enterotypes, and the taxa with the highest relative abundances among these differential microorganisms were regarded as the proxy of the enterotypes (Huang et al., 2022). Values were represented as the mean ± standard error of the mean (SEM) unless otherwise noted. Statistical significance was set at a *p*-value of < 0.05.

## Results

### Fecal microbiota characteristics in small ruminants among different herds

**Figure 1:**
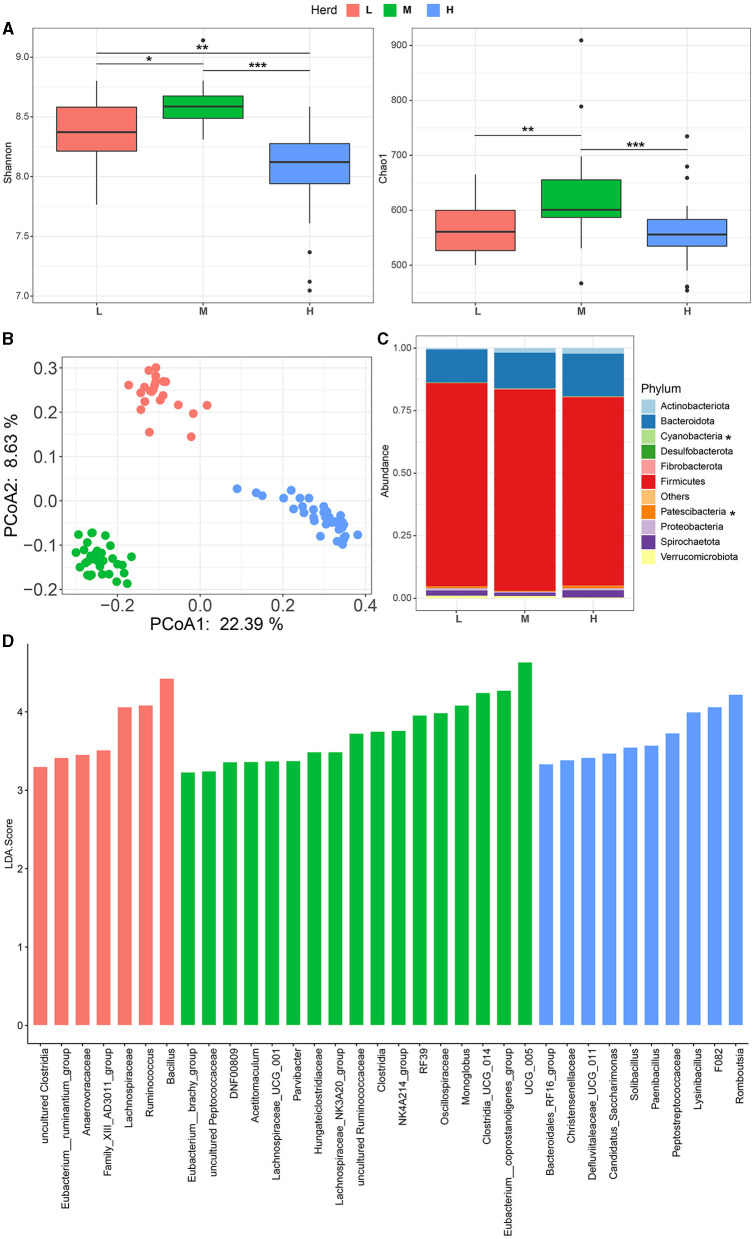
Fecal microbial community composition in small ruminants among different herds. Alpha **(A)** and Beta **(B)** diversities at different herds. **(C)** Fecal bacterial composition at the phylum level. **(D)** Differential fecal bacterial taxa within different herds. * *p*< 0.05, ** *p*< 0.01, and *** *p*< 0.001. L, Qianbei Ma goats; M, Hei goats; H, Weining sheep.

After quality control, a total of 1,794,289 sequences with an average of 19,936 sequences per sample were obtained and assigned to 8,564 ASVs (Table 1). The Good's coverage for all animals was found to be over 99.97%, indicating that the sequencing depth covered most of the fecal bacteria. The Shannon and Chao1 indices were higher in the M herd among the three herds (Figure 1A), and the bacterial community displayed a clear separation based on the herd under study (PERMANOVA *P* = 0.001, Figure 1B).

**Table 1:** The detailed information of the sample of different herds^a^ and sequence features obtained using the DADA2 algorithm.

**Herd**	**Sex**	**Number of samples**	**Number of sequences**	**Frequency**	**Number of ASVs**
L	Female	11	55,513 ± 504	18,701 ± 286	561 ± 12
	Male	12	54,209 ± 645	18,490 ± 227	569 ± 16
M	Female	16	52,822 ± 2,098	20,016 ± 842	608 ± 17
	Male	16	53,031 ± 1,799	20,049 ± 836	641 ± 21
H	Female	23	54,075 ± 1,282	21,229 ± 682	565 ± 13
	Male	12	52,261 ± 980	19,783 ± 286	544 ± 13

^a^L, Qianbei Ma goats; M, Hei goats; H, Weining sheep.

For the taxonomic profile, a total of 11 phyla were identified according to the selected criteria mentioned in the Materials and Methods section, with Firmicutes (78.7 ± 0.01%) and Bacteroidota (14.8 ± 0.01%) (synonym Bacteroidetes) being the predominant taxa in the fecal microbiome (Figure 1C, Supplementary Table S1). The relative abundances of Cyanobacteria and Patescibacteria were significantly different between different herds (*p* < 0.05, Figure 1C). In total, 108 genera were identified based on the selected criteria, of which *UCG-005* (12.1 ± 0.005%) was the dominant genus, followed by *Christensenellaceae_R-7_group* (11.2 ± 0.004%), f.*Lachnospiraceae* (5.7 ± 0.002%), *UCG-010* (3.7 ± 0.001%), *Clostridia_UCG-014* (3.4 ± 0.002%), and *Rikenellaceae_RC9_gut_group* (3.0 ± 0.002%) (Supplementary Table S2). Among these, a total of 34 bacterial genera were differentially identified among different herds (Figure 1D). Specifically, the fecal microbiota of the L herd was enriched with *Bacillus* (L: 5.1%, M: 0, and H: 1.1%) and *Ruminococcus* (L: 3.7%, M: 1.9%, and H: 1.4%) (Figure 1D, Supplementary Table S2). Moreover, the relative abundances of *UCG_005* (M: 16%, L: 13.1%, and H: 7.9%) and *Clostridia_UCG_014* (M: 5.2%, L: 3.3%, and H: 1.9%) were found to be overrepresented in the M herd (Figure 1D, Supplementary Table S2). In addition, the relative abundances of *Romboutsia* (H: 4.5%, L: 1.5%, and M: 2.3%) and *F082* (H: 2.4%, L: 0.3%, and M: 0.2%) were greater in the H herd (Figure 1D, Supplementary Table S2). The Venn diagram showed that 51 genera were shared by all herds and that the L herd and M herd shared more taxa with each other than with the H herd. Each herd had unique taxa, and the number of unique genera in the H herd (*n* = 17) was greater than that in the M herd (*n* = 8) and the L herd (*n* = 9) (Supplementary Figure S1).

### Effect of sex on the composition of the fecal microbiota

**Figure 2:**
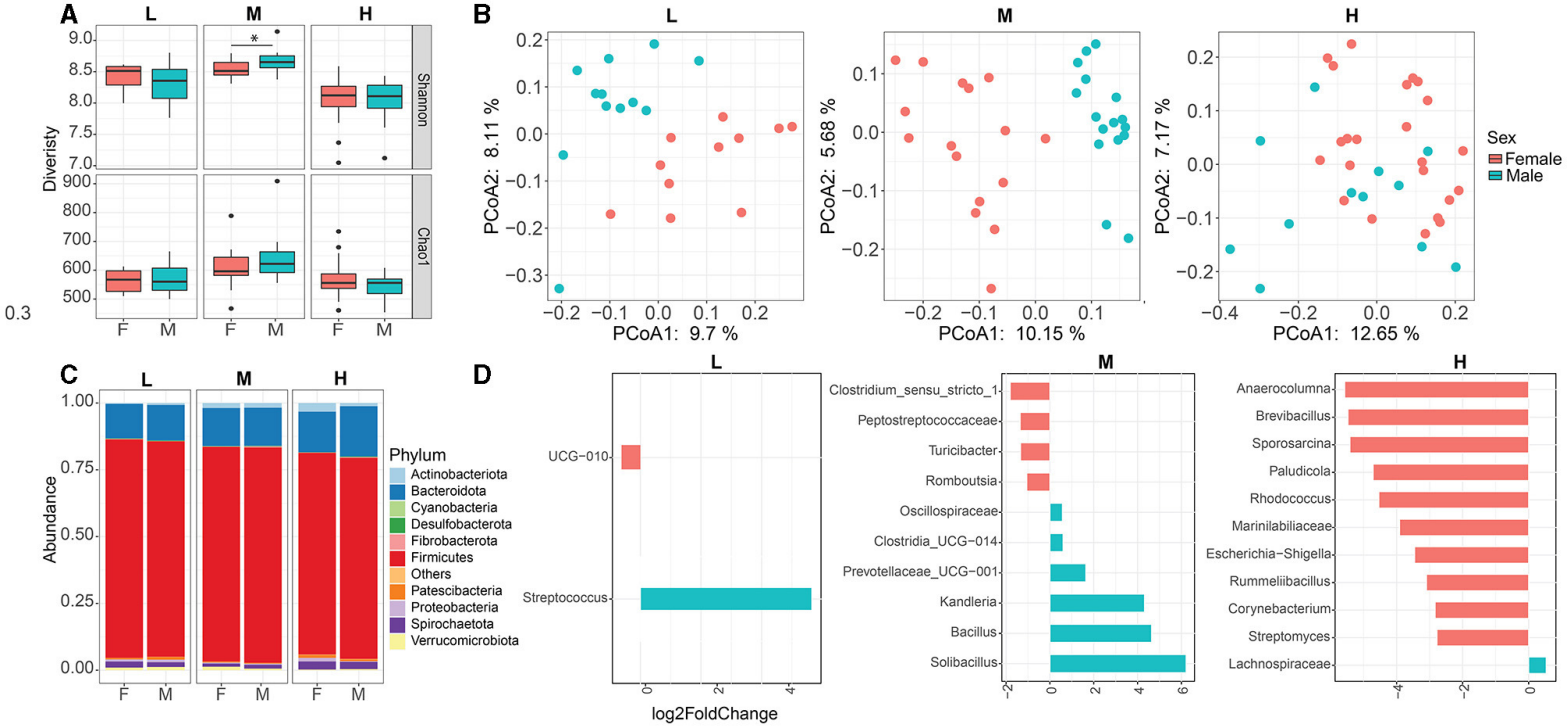
Effect of sex on fecal microbial community composition within each herd. Alpha **(A)** and Beta **(B)** diversities between female and male animals within each herd. **(C)** Fecal microbial composition between female and male animals at the phylum level. **(D)** Differential microbial taxa between female and male animals within each herd. * *p*< 0.05. L, Qianbei Ma goats; M, Hei goats; H, Weining sheep.

To determine whether sex affects the fecal microbiota profile, the microbial composition between female and male animals within each herd was compared. The alpha diversity (Shannon and Chao1) indices did not differ between the female and male animals in the L herd and H herd, whereas the Shannon index was greater in male animals than in female animals in the M herd (Figure 2A). The principal coordinate analysis (PCoA) based on Bray–Curtis distances displayed a clear separation between female and male animals within each herd (PERMANOVA *p* < 0.05, Figure 2B).

Taxonomic profiles were analyzed to evaluate and determine variations in the bacterial structure between female and male animals within each herd. Overall, the dominant phyla were Firmicutes (female vs. male 80.4% vs. 80.6%), Bacteroidota (female vs. male: 14.2% vs. 14.3%), and Actinobacteriota (female vs. male: 1.9% vs. 1.8%) in the M herd, and the relative abundances of Fibrobacterota (female vs. male: 0.1% vs. 0.4%) and Desulfobacterota (female vs. male: 0.07% vs. 0.1%) were greater in male animals than in female animals (*p* < 0.05). In addition, the relative abundance of Verrucomicrobiota (female vs. male: 1.3% vs. 0.6%) was greater in female animals than in male animals in the M herd. Similarly, the predominant phyla in the L herd and H herd were Firmicutes (L: 81.8% vs. 80.6%; H: 75.5% vs. 75.2%), Bacteroidota (L: 12.9% vs. 13.3%; H: 15.2% vs. 18.8%), and Actinobacteriota (0.35% vs. 0.75%). Patescibacteria (0.46% vs. 0.98%) were lesser in female animals than in male animals in the L herd (*p* < 0.05, Figure 2C). Furthermore, the relative abundance of Actinobacteriota was greater in female animals than in male animals in the H herd (Figure 2C). At the genus level, the top four most abundant genera were *UCG-005, Christensenellaceae_R-7_group, Lachnospiraceae*, and *UCG-010* within each herd (Supplementary Table S2), and the relative abundances of *Lachnospiraceae* and *UCG-010* were distinct between female and male animals from the L herd and H herd (Figure 2D).

### Enterotype profile of bacterial community

**Figure 3:**
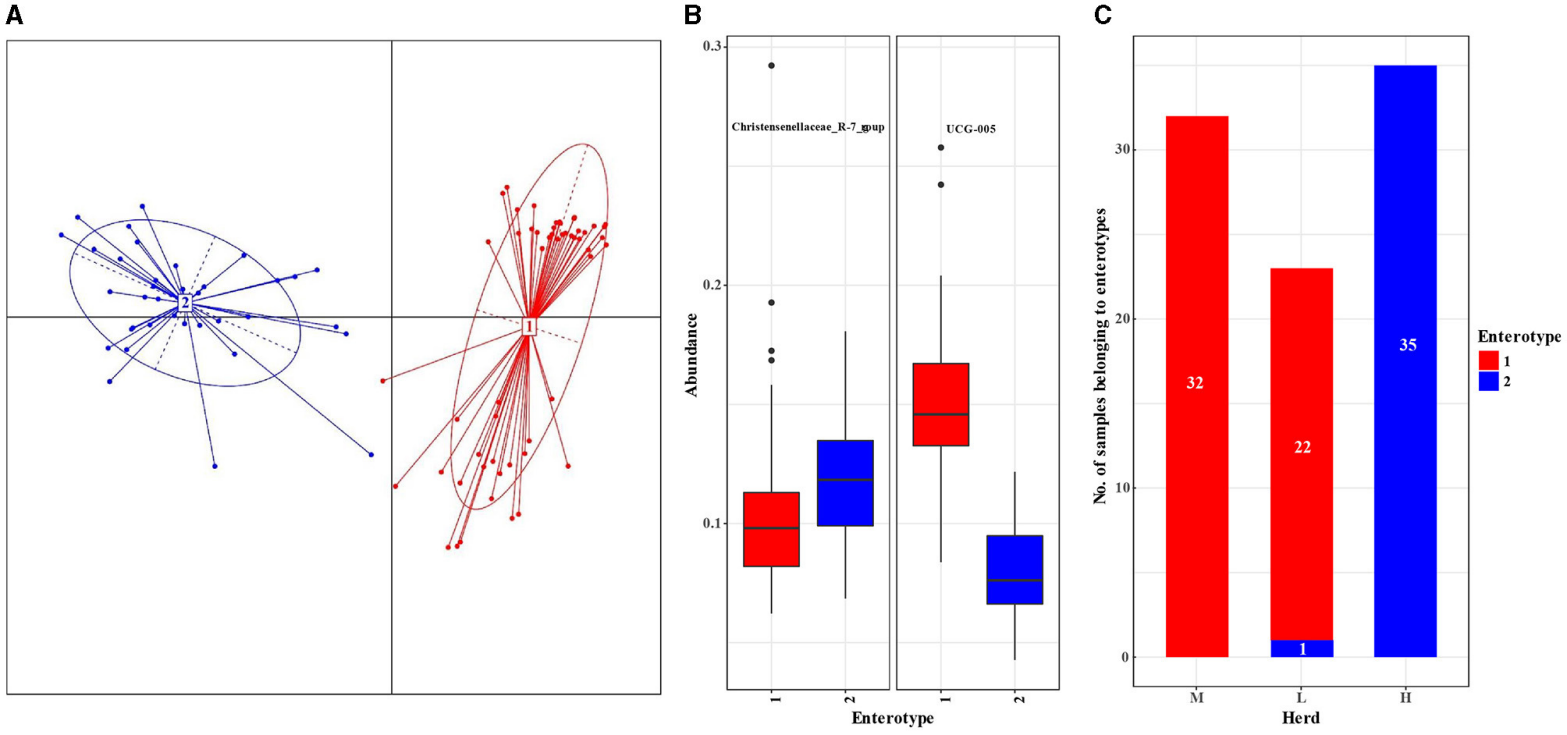
Analysis of enterotypes in the fecal microbiota in small ruminants among different herds. **(A)** Principal coordinate analysis plot based on the Jensen–Shannon divergence distance at the genus level. **(B)** Relative abundances of representative genera in each enterotype. **(C)** Number of samples belonging to the enterotypes of each herd.

A total of 90 fecal samples were divided into enterotypes 1 and 2 using principal coordinate analysis (PCoA) (Figure 3A), and the enterotypes were mainly driven by genera *UCG-005* (enterotype 1, *n* = 54) and *Christensenellaceae_R-7_group* (enterotype 2, *n* = 36) (Figure 3B), respectively. All samples from the M herd and 22 samples from the L herd belonged to enterotype 1, whereas all samples from the H herd and 1 sample from the L herd belonged to enterotype 2 (Figure 3C).

**Figure 4:**
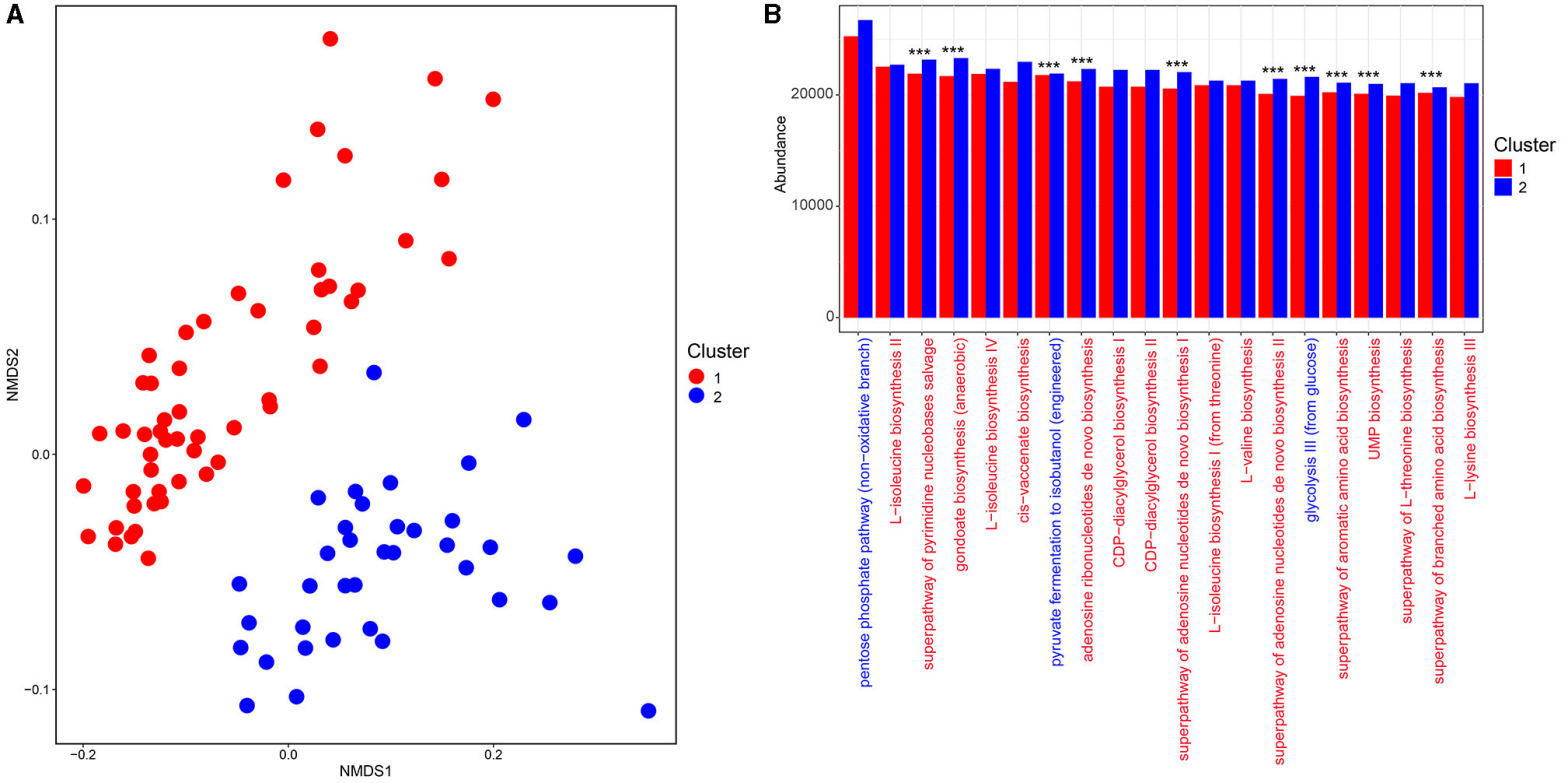
Comparison of functional pathways between enterotype groups. **(A)** The non-metric multidimensional scaling (NMDS) plot of the functional profile of the fecal bacterial communities between enterotypes 1 and 2 based on the Bray–Curtis distances. **(B)** The top 20 ranked MetaCyc pathways between enterotypes 1 and 2. The red font represents the MetaCyc pathways belonging to biosynthesis, while the blue font represents the MetaCyc pathways belonging to the generation of precursor metabolites and energy. *** *p*< 0.001.

The fecal bacterial community functions of enterotypes 1 and 2 were predicted and stratified, and the metabolic pathway abundances were investigated. Non-metric multidimensional scaling (NMDS) based on Bray–Curtis distances was used to depict the clustering of metabolic functional profiles, and two functional clusters (1 and 2) that corresponded to enterotypes 1 and 2 were obtained (ANOSIM R = 0.57, *p* < 0.001, Figure 4A). A total of 371 MetaCyc metabolic pathways potentially encoded by fecal bacteria were predicted, of which 211 metabolic pathways were differentially represented between clusters 1 and 2, with 92 pathways being enriched in cluster 1 and 119 in cluster 2 [Linear discriminant analysis (LDA) > 2, *p* < 0.05, Supplementary Table S3]. Moreover, the abundances of the top 20 ranked metabolic pathways belonged mainly to biosynthesis (amino acid biosynthesis, fatty acid and lipid biosynthesis, and nucleoside and nucleotide biosynthesis), while only three metabolic pathways mapped to the generation of precursor metabolites and energy (glycolysis, pentose phosphate, and fermentation pathways) (Figure 4B). Of these, 10 were overrepresented in cluster 2 (Figure 4B).

## Discussion

Small ruminants play an important role in the global food economy, and their fecal microbiota play key roles in host metabolism and productivity performance. However, considering the importance of fecal microbial composition to the host, few studies have been conducted to explore it and its potential influential factors on indigenous small ruminants in the Chinese province of Guizhou. This study provides a comprehensive overview of the fecal bacterial microbial profiles across three local ruminant breeds (two goat breeds and one sheep breed) living in different areas of Guizhou and demonstrates that fecal microbial communities differ according to sex and herd (genetics/altitude). Furthermore, two enterotypes were identified, with enterotype 1 dominated by *UCG-005* and enterotype 2 by *Christensenellaceae_R-7_group*. In addition, the predicted functional profiles of the fecal microbiota were divided into two functional clusters that corresponded to the identified enterotypes.

In the present study, the Shannon diversity was significantly different among ruminant species. This is consistent with previous reports on other ruminant species (O'Donnell et al., 2017; Chang et al., 2020), where the diet varied across species. However, a recent study reported that there was no significant difference in the alpha diversity of fecal microbiota between sheep and goats when they were fed the same diet (Shabana et al., 2021). This discrepancy suggests that diet supersedes host species in shaping the fecal microbiota (Wei et al., 2021). Moreover, the Firmicutes/Bacteroidota ratio varied across species. It was reported that Firmicutes and Bacteroidetes are involved in the digestion of carbohydrates and proteins, and the Firmicutes/Bacteroidota ratio is positively associated with average daily gain (ADG) and the milk fat composition in bovines (Maslen et al., 2023). In addition, host-specific taxa were found in each ruminant species. For instance, *Bacteroidales_BS11_gut_group* was detected uniquely in the H herd, *Succiniclasticum* was detected uniquely in the L herd, while *Olsenella* was detected uniquely in the M herd. *Bacteroidales_BS11_gut_group* specializes in fermenting hemicellulose monomeric sugars and acetate production (Solden et al., 2017). *Succiniclasticum*, a type of amylolytic bacteria, possesses the ability to convert succinate to propionate (Zhang et al., 2018). *Olsenella* could produce lactic acid from glucose fermentation (Gaowa et al., 2021). Thus, we hypothesized that variations in the composition of fecal microbiota among different species may contribute to the differences in the feed utilization of the host since fecal microbiota are host-specific and associated with host phenotypes (Mallott and Amato, 2021; Andrade et al., 2022).

In addition, the Shannon diversity was significantly higher in the L herd (low altitude) and M herd (median altitude) compared to the H herd (high altitude), which is consistent with a previous study on Tibetan humans and pigs (Zeng et al., 2020). However, a recent study on mammals (macaques, humans, and dogs) found that Shannon diversity was higher in high-altitude populations than in their low-altitude counterparts (Zhao et al., 2023), and similar observations were also seen in mice under simulated high-altitude environments (Wang F. et al., 2022) and in Tibetans (Lan et al., 2017). These controversial results indicate that altitude is not the only driver of fecal microbiota alpha diversity since genetic background, life environments, and diet also act as the deterministic drivers in shaping it (Zeng et al., 2020). Recent studies have reported that the profile of fecal microbiota is mainly influenced by altitudes in indigenous animals (Ma et al., 2019) and that the yak fecal microbial community shows a clear clustering of taxa according to altitudes (Liu et al., 2021). Thus, we speculated that variations in altitude may have contributed to the differences in the fecal bacterial community of the small ruminants in the current study. Firmicutes and Bacteroidota were the most abundant phyla in the fecal microbiota across altitudes; this finding is supported by the results of the previous studies on ruminants (Lei et al., 2018; Cui et al., 2019; Liu et al., 2019), highlighting that they are essential components of the fecal microbiota in ruminants. In this study, the Firmicutes/Bacteroidota ratio showed a noticeable downward trend in the H herd (high altitude) compared to the L herd (low altitude) and the M herd (medium altitude). However, multiple studies have consistently indicated that small ruminants living at high altitudes, such as Tibetan antelope, European mouflon, and blue sheep, exhibit a higher Firmicutes/Bacteroidota ratio compared to their low-altitude counterparts (Ma et al., 2019; Sun et al., 2019). Moreover, it has been demonstrated that the Firmicutes/Bacteroidota ratio influences cardiorespiratory fitness (Durk et al., 2019) and is treated as an indicator of gut dysbiosis (Yu et al., 2019). In addition, the Firmicutes/Bacteroidota ratio changed with diet (Zhang et al., 2018b) and was positively associated with energy harvest (Wang F. et al., 2022). Furthermore, the high ratio of Firmicutes to Bacteroidetes was beneficial to the utilization of plant cellulose in pikas (Li et al., 2016). Based on these findings, we postulated that changes in the ratio of Firmicutes to Bacteroidota across altitudes facilitates a high-altitude adaptation of the host (Wang X. et al., 2022), which may be attributed to noticeable differences in dietary habits (Li and Zhao, 2015).

At the genus level, the relative abundances of *Romboutsia, Lysinibacillus, Peptostreptococcaceae, Christensenellaceae, Defluviitaleaceae_UCG_011, Candidatus_Saccharimonas, Paenibacillus*, and *Solibacillus* were greater in the H herd (high altitude) than in other herds (low and medium altitudes). The microbes that are overrepresented in the high-altitude area probably perform important functions for the host. For instance, members of *Lysinibacillus* isolated from the Taihu Lake are iron-reducing strains (Li et al., 2023), and hypoxia signaling links erythropoiesis with iron homeostasis (Renassia and Peyssonnaux, 2019). Furthermore, *Paenibacillus* spp. can produce a variety of exopolysaccharides (EPSs) that have been proven to have antioxidative properties, and antioxidants play a crucial role in the adaptation of animals to high-altitude environments (Zeng et al., 2017). In addition, *Paenibacillus polymyxa 10* could enhance the growth performance of broilers by improving intestinal health (Wang et al., 2021). *Christensenellaceae* contributes to the host health by modulating lipid metabolism (Shen et al., 2021). Moreover, *Solibacillus* acts as probiotics against pathogens, such as *Aeromonas* and *Pseudomonas* (Ayoola et al., 2023). Taken together, these potential probiotics in the H herd may help animals improve metabolic function and maintain intestinal homeostasis to adapt to high-altitude environments. More research is warranted to substantiate the ecological functions of these bacteria in high-altitude small ruminants. Contrary to the observed potential probiotics, two potential pathogenetic bacteria were also enriched in the H herd. *Romboutsia* functions as a potential pathobiont in ulcerative colitis (Wang F. et al., 2022), while *Defluviitaleaceae_UCG_011* is positively associated with intestinal dysbiosis (Yang et al., 2023). These results suggest that high altitude may increase the number of pathogenic bacteria that induce intestinal dysfunction (Wan et al., 2022) and climate change may lead to wider spreading of pathogens (Rocklöv and Dubrow, 2020). The relative abundances of fiber-degrading bacteria such as *Bacillus* and *Ruminococcus* (Pandit et al., 2018) were enriched in the L herd (low altitude) compared to other herds. *Ruminococcus* converts cellulose, hemicellulose, and other polysaccharides into acetate and succinate (La Reau and Suen, 2018) and is positively associated with fumaric acid concentration that inhibits rumen methane production (Hu et al., 2020). *Bacillus* produces amino acids, stimulates protein synthesis, and is a major reservoir of lipolytic enzymes (Mukendi et al., 2018). In this study, the relative abundance of *UCG_005* was greater in the M herd (medium altitude) than in the other herds. Members of this genus enhance fiber digestion (Yang et al., 2020) and are related to the production of ruminal acetate and total short-chain fatty acids (SCFAs) (Li et al., 2021). In addition, *UCG_005* produces butyric acids that enhance the intestinal surface area, improving calcium absorption (Zhou et al., 2023). The differentially abundant profile in fecal bacteria across altitudes suggests that the fecal microbiota follow a divergent pattern in functional adaptation according to the external environment, genetic background, or diet resources (Zhang et al., 2018b).

In the present study, the fecal microbiota composition differed between male and female animals within each herd, which is consistent with the findings of a recent study on fecal microbiota composition in different ruminant species on the Qinghai-Tibetan Plateau that identified sex as one of the key factors influencing the fecal microbiota composition (Wang X. et al., 2022). Sex-related differences in fecal microbiota composition were also observed in Hanwoo cattle (Sim et al., 2022), Chinese forest musk deer (Zhao et al., 2019), and Dromedary camels in Saudi Arabia (Elbir and Alhumam, 2022). In this study, a greater abundance of *UCG*−*010* in male animals and a greater abundance of *Streptococcus* in female animals in the L herd were observed. *UCG*−*010* is crucial for fiber degradation (mainly in cellulose digestion) and biohydrogenation process of converting dietary polyunsaturated fatty acids (PUFAs) to saturated fatty acids (SFAs) (Guerra et al., 2022). *Streptococcus* is a key probiotic in ruminant feedings (Kulkarni et al., 2022), and a bacteriocin produced by *Streptococcus bovis* can reduce methane emissions from ruminants (Lee et al., 2002). In addition, the abundance of *Anaerocolumna* was higher in female animals than in male animals, whereas *Lachnospiraceae* was greater in male animals than in female animals in the H herd. *Anaerocolumna* has the potential to degrade lignin and chitin (Ueki et al., 2019; Xu et al., 2021), while *Lachnospiraceae* plays an important role in fiber digestion (Yang et al., 2020). These findings suggest that the fecal microbiota community varies with sex, which may be ascribed to different sex hormones (Kim, 2023) and diet preferences between male and female animals (Lee et al., 2017).

The predicted outcomes of the bacterial community structure (enterotype) can be highly dependent on host genetics rather than on altitude and the sex of the collected sample, as demonstrated in the current study. This finding is in contradiction with previous studies on other ruminant species and humans, where the enterotypes were diet-driven (Wu et al., 2011; Hicks et al., 2018; Couch et al., 2021). However, it has been demonstrated that host genetics is an important contributor to the enterotype of individual fecal microbiota in humans and pigs (Lim et al., 2014; Ma et al., 2022), and the gut microbiome is host-specific (Mallott and Amato, 2021). Thus, the division of fecal microbiota into different enterotypes between goats and sheep could be expected. In this study, enterotype 1 and enterotype 2 were characterized by *UCG-005* and *Christensenellaceae_R-7_group*, respectively. Consistent with our observation of the enrichment of biosynthesis pathways (i.e., amino acid biosynthesis and fatty acids) in enterotype 1, species of *UCG-005* are associated with acetate and SCFA production and increased intestinal absorption of nutrients (Li et al., 2021; Zhou et al., 2023). In addition, the pathways associated with energy metabolism (glycolysis II from fructose 6-phosphate, glycolysis I from glucose 6-phosphate, and aerobic respiration I cytochrome c) were enriched in enterotype 2. *Christensenellaceae_R-7_group* plays an important role in degrading carbohydrates and amino acids to acetate and ammonia, with acetic and butyric acids as fermentation end products (Chen et al., 2020), contributing to the enrichment of energy metabolism pathways in enterotype 2. In our study, the pathways associated with energy metabolism were significantly enriched in the H herd (high altitude) compared to the other herds. This finding is supported by a study on Tibetans and Tibetan pigs, where the relative abundances of energy metabolism pathways were significant in high-altitude groups (Zeng et al., 2020). These findings suggest that fecal microbiota helps the host in adapting to high-altitude energy requirements by modulating relevant metabolic functions.

One of the limitations of the current study was the lack of data on blood oxygen levels. It was reported that blood oxygen level is an important factor that affects the intestinal microbial community (Zhang et al., 2018a) via maintaining the balance between aerobic and anaerobic environments in the intestinal epithelium (Bai et al., 2022); thus, data on blood oxygen level should be taken into consideration in future studies. Furthermore, samples from different ruminant species at the same altitude were not collected. Future studies with more and larger-scale samples from different ruminant species at the same altitude (below 1,000 m and above 3,000) are warranted to verify the findings in this study.

In conclusion, this study characterized bacterial fecal microbiota in indigenous small ruminants living in different areas of Guizhou. The microbiota were clustered into two enterotypes led by *UCG-005* (E1) and *Christensenellaceae_R-7_group* (E2), which were highly dependent on host genetics (breeds), although altitude and sex also contributed to the differences in the fecal microbiota community. In addition, the fecal microbiota enterotypes corresponded to the clustering of functional prediction profiles, confirming the existence of these two enterotypes in our animal cohort. These results provide detailed and novel insights into the composition, function, and enterotypes in the fecal microbiota of local sheep and goats in Guizhou, which may help us understand how fecal microbiota contribute to different host phenotypes in indigenous small ruminants and their adaptation mechanism in high-altitude environments.

## Data availability statement

The datasets presented in this study can be found in online repositories. The names of the repository/repositories and accession number(s) can be found below: https://www.ncbi.nlm.nih.gov/, PRJNA1006184.

## Ethics statement

The animal study was approved by the Animal Ethics Committee of Guizhou University. The study was conducted in accordance with the local legislation and institutional requirements.

## Author contributions

WG: Conceptualization, Formal analysis, Writing – original draft, Writing – review & editing. TL: Data curation, Visualization, Writing – review & editing. WW: Formal analysis, Methodology, Writing – review & editing. YY: Methodology, Software, Writing – review & editing. AN: Conceptualization, Supervision, Visualization, Writing – review & editing. MZ: Conceptualization, Supervision, Validation, Visualization, Writing – review & editing. XC: Funding acquisition, Project administration, Writing – review & editing.
