# Supplementary material for: Survey of the fecal microbiota of indigenous small ruminants living in different areas of Guizhou
Source: Front Microbiol. 2024 Aug 8;15:1415230. doi: 10.3389/fmicb.2024.1415230 (PMC11340823; doi:10.3389/fmicb.2024.1415230)
Supplement: Supplementary file 2 [file Data_Sheet_1.docx]

**Supplementary Figure**

**
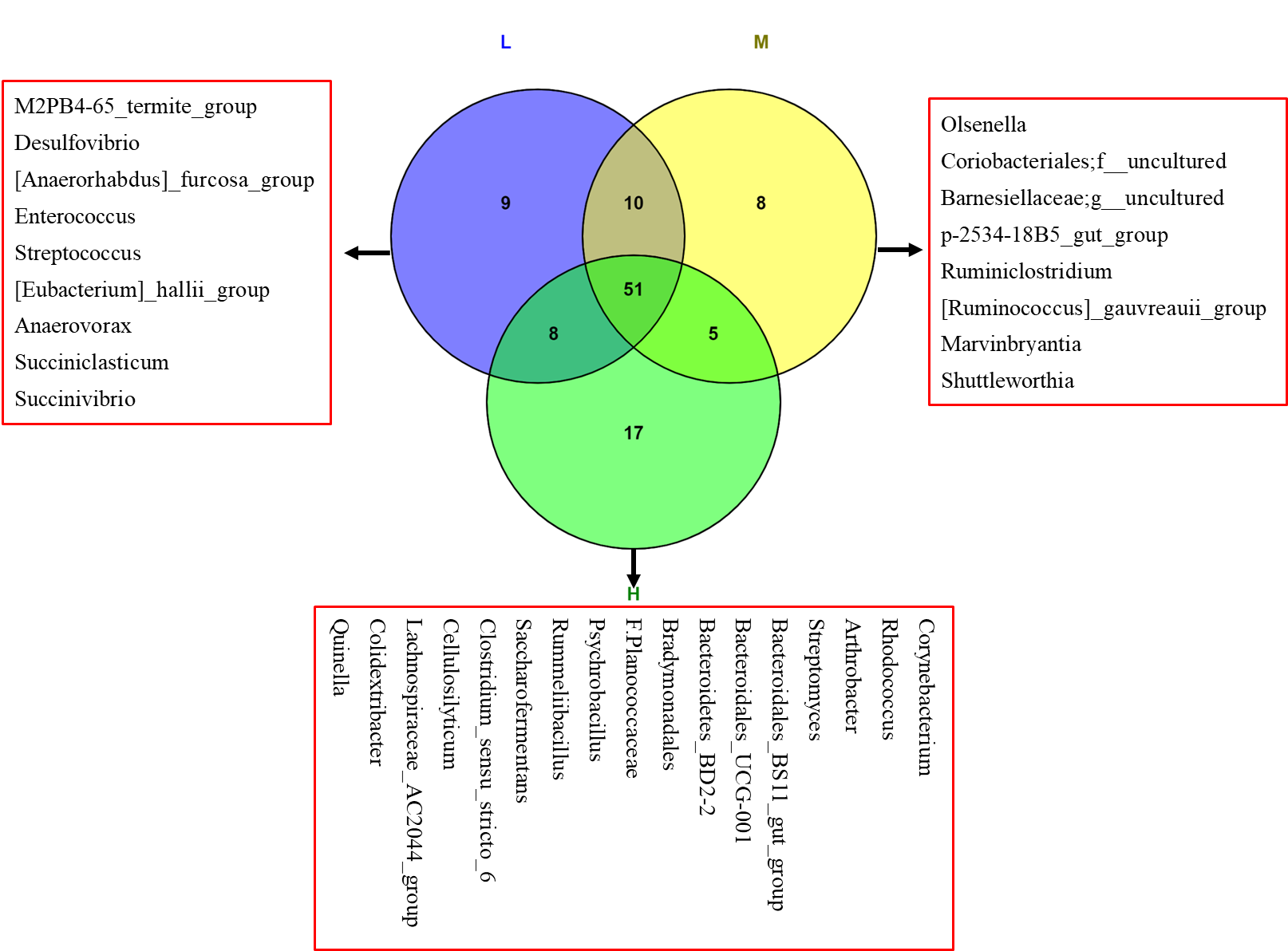
**

**Supplementary Figure 1.** Venn diagram displaying the shared and unique genera in small ruminants across herds. L, Qianbei Ma goats; M, Hei goats; H, Weining sheep.
